# A look at the potential association between PICOT framing of a research question and the quality of reporting of analgesia RCTs

**DOI:** 10.1186/1471-2253-13-44

**Published:** 2013-11-19

**Authors:** Victoria Borg Debono, Shiyuan Zhang, Chenglin Ye, James Paul, Aman Arya, Lindsay Hurlburt, Yamini Murthy, Lehana Thabane

**Affiliations:** 1 Department of Anesthesia, McMaster University, 2U1-1200 Main Street West, Hamilton, Ontario L8N 3Z5, Canada; 2 Department of Clinical Epidemiology and Biostatistics, McMaster University, 1200 Main Street West, Hamilton, Ontario L8N 3Z5, Canada; 3 Biostatistics Unit, Father Sean O’Sullivan Research Centre, 3rd Floor Martha, Room H325, St. Joseph’s Healthcare Hamilton, 50 Charlton Avenue East, Hamilton, Ontario L8N 4A6, Canada; 4 Department of Medicine, Schulich School of Medicine, University of Western Ontario, 339 Windermere Road, London, Ontario N6G 2 K3, Canada; 5 Department of Anesthesia, University of Toronto, Room 121, Fitzgerald Building, 150 College Street, Toronto, Ontario M5S 3E2, Canada

## Abstract

**Background:**

Methodologists have proposed the formation of a good research question to initiate the process of developing a research protocol that will guide the design, conduct and analysis of randomized controlled trials (RCTs), and help improve the quality of reporting such studies. Five constituents of a good research question based on the PICOT framing include: Population, Intervention, Comparator, Outcome, and Time-frame of outcome assessment. The aim of this study was to analyze if the presence a structured research question, in PICOT format, in RCTs used within a 2010 meta-analysis investigating the effectiveness of femoral nerve blocks after total knee arthroplasty, is independently associated with improved quality of reporting.

**Methods:**

Twenty-three RCT reports were assessed for the quality of reporting and then examined for the presence of the five constituents of a structured research question based on PICOT framing. We created a PICOT score (predictor variable), with a possible score between 0 and 5; one point for every constituent that was included. Our outcome variable was a 14 point overall reporting quality score (OQRS) and a 3 point key methodological items score (KMIS) based on the proper reporting of allocation concealment, blinding and numbers analysed using the intention-to-treat principle. Both scores, OQRS and KMIS, are based on the Consolidated Standards for Reporting Trials (CONSORT) statement. A multivariable regression analysis was conducted to determine if PICOT score was independently associated with OQRS and KMIS.

**Results:**

A completely structured PICOT score question was found in 2 of the 23 RCTs evaluated. Although not statistically significant, higher PICOT was associated with higher OQRS [IRR: 1.267; 95% confidence interval (CI): 0.984, 1.630; p = 0.066] but not KMIS (1.061 (0.515, 2.188); 0.872). These results are comparable to those from a similar study in terms of the direction and range of IRRs estimates. The results need to be interpreted cautiously due to the small sample size.

**Conclusions:**

This study showed that PICOT framing of a research question in anesthesia-related RCTs is not often followed. Even though a statistically significant association with higher OQRS was not found, PICOT framing of a research question is still an important attribute within all RCTs.

## Background

In a previous study we recently completed, we found poor overall quality of reporting [1] with randomized controlled trials (RCTs) used in a femoral nerve block meta-analysis [2]. We also identified considerable shortcoming in the reporting of three key methodological items with these RCTs. Most of the articles used in the meta-analysis came from journals specialized in anesthesia. Poor quality of reporting of RCTs is not exclusive to medical journals specialized in anesthesiology literature [3]. Similar findings have been reported in major general medical journals and subspecialty journals [4-15].

The lack of transparency in RCT reporting greatly reduces the readers’ ability to judge the quality, validity and reliability of the findings. It is difficult for the reader to find information in a study when reporting of certain qualities, especially those specified by the CONSORT 2010 statement [16], is done in a vague manner. Inadequate reporting and design of RCTs are associated with bias, especially exaggerated intervention or treatment effects that influence the interpretation of the findings of RCTs in helping to develop clinical guidelines and in being used for meta-analyses [16-18]. Formal critical appraisal of trials is much more feasible with high quality of reporting of studies. This is because the published paper is typically the primary gateway for most readers to review RCTs. Although it is acceptable practice to contact trial authors to obtain data and other study details, the published article is the most accessible dissemination of the research that can be evaluated. The Consolidated Standards of Reporting Trials (CONSORT) group have published updated guidelines in the CONSORT 2010 statement that provide guidance on the reporting of RCTs for authors and for the medical publishing community at large [16-18]. This information is available at http://www.consort-statement.org[18]. Though there have been some significant improvements in the quality of reporting since the CONSORT statement publication in 2001, the quality of reporting remains well below acceptable [10].

It is possible that certain predictor variables are associated with increased quality of reporting. Journal impact factor, sample size and declared funding have been associated with better quality of RCT reporting [3,6,11,15,19,20]. Additionally, journal adoption of the CONSORT statement has been associated with improved reporting of RCTs [6,16,21-23]. Being able to identify variables which can act as predictors for RCT quality of reporting could better help medical practitioners to review current literature; including literature within anesthesia and pain management. In an observational study it was found that journal publication, type of funding (particularly complete industrial funding) and larger sample sizes were significantly associated with a slightly better quality of reporting score [3]. It is also possible that better framing of a research question within an RCT can be independently associated with better overall quality of reporting and reporting of key methodologies, as were reported [24].

Methodologists have proposed the formation of a good research question to maximize the process of developing a research protocol, the study design and analytical assessments needed for a study being undertaken [25,26]. A good question can aid an investigator tremendously with choosing the correct methodology and strategy for analysis, which in turn helps with appropriately answering the primary research question [25-27]. A good research question is structured and requires the specification of the five constituents according to the PICOT approach [28]. These five constituents are: Population, Intervention, Comparator, Outcome, and Time-frame of outcome assessment (or commonly known by the acronym PICOT) [25,26,28,29]. The PICOT approach is clear, concise and easy to use in terms of framing all the components of a research question [25]. The use of a properly structured research question has been proposed to guide the process of developing the research design and protocol [25-27]. Our hypothesis is that a clear and complete research question that contains all the PICOT elements would spur better quality of reporting. The rationale behind this hypothesis is that a more complete PICOT question would drive the formation of a thorough research methodology to fully address the specifics of the research question and hence motivate detailed reporting of what was done in the study. More evidence is still needed to help the medical research community better understand whether adherence to the PICOT approach to developing a research question is associated with an increase in the quality of reporting in research publications. The aim of this observational study was to determine if the presence of a structured research question in PICOT format is independently associated with improved quality of reporting of RCTs. In this study, we quantified quality of reporting using a quality of reporting scoring system, which is described in the Methods.

## Methods

### Study design

This is an observational study based on 23 RCTs used in a meta-analysis to compare the analgesia outcomes of femoral nerve block (FNB), with or without sciatic nerve block, with epidural or patient controlled analgesia (PCA) after total knee arthroplasty [2]. The 23 RCTs used in the above mentioned meta-analysis were assessed in a previous study we conducted [1]. The following two things were assessed for in this earlier study: 1) the quality of reporting using a 15 point overall quality of reporting score (OQRS) [1], 2) key methodological item score (KMIS) using a 3 point score [1]. In this study, we went on to assess these same 23 RCTs used in the FNB meta-analysis [2] for a structured research question using the PICOT format with a 5 point score. The first two quality assessments of the 23 RCTs in the FNB meta-analysis [2], OQRS and KMIS, were, as mentioned above, done in a prior study that evaluated the quality of reporting and reporting of methodological items as the outcome variables as well as four predictor variables hypothesized to correlate with better outcomes (OQRS and KMIS) [1]. The data collected from this previous study was used to examine if a structured research question using the PICOT format is associated with better OQRS and KMIS in the same 23 RCTs [1]. New data collected for this study was in relation to variables needed to assess the presence of a structured research question within the 23 RCTs, using the PICOT format (Population, Intervention, Comparator, Outcome, Timeframe) with a 5 point score (score out of 5, with a possible range of score from 0 to 5 inclusively). For the purpose of this study, our original 15 point OQRS was reduced to a 14 point score because one of the 15 items we initially assessed for in our previous study [1] was “objectives” and this was in part assessed as part of our PICOT score (item 6 on Table 1). Much of our study’s method and protocol design models another study that also looked at the association between a PICOT structured research question and quality of reporting and reporting of key methodological items in RCTs, separately [24]. We found this study by Rios *et al.*[24] to have a sound methodology for effectively evaluating important variables needed to assess the quality of reporting, key methodological items in RCTs and a structured research question using the PICOT format.

**Table 1:** Description of overall quality of reporting items

**Item**	**Description**^**a**^
1. Title or Abstract	Identification as a randomized trial in the title and a structured summary of the trial design, methods, results, and conclusions.
**Introduction**	
2. Background	Scientific background and explanation of rationale.
3. Objectives	Specific objectives or hypotheses.
**Methods**	
4. Participants	Eligibility criteria for participants.
	Settings and locations where the data were collected
5. Interventions	The interventions for each group with sufficient details to allow replication, including how and when they were actually administered.
6. Outcomes*	Completely defined pre-specified primary and secondary outcome measures, including how and when they were assessed.
7. Sample Size	How sample size was determined.When applicable, explanation of any interim analyses and stopping guidelines.
8. Randomization: Sequence Generation	Method used to generate the random allocation sequenceType of randomisation; details of any restriction (such as blocking and block size).
9. Randomization: Implementation	There is mention of: Who generated the random allocation sequence, who enrolled participants, and who assigned participants to interventions?
10. Statistical Methods	Statistical methods used to compare groups for primary and secondary outcomes.
	Methods for additional analyses, such as subgroup analyses and adjusted analyses.
**Results**	
11. Participants Flow	For each group, the numbers of participants who were randomly assigned, received intended treatment, and were analysed for the primary outcome.
	For each group, losses and exclusions after randomisation, together with reasons.
12. Recruitment	Dates defining the periods of recruitment and follow-up
	Why the trial ended or was stopped.
13. Baseline Data	A table showing baseline demographic and clinical characteristics for each group.
14. Outcomes and Estimates	For each primary and secondary outcome, results for each group, and the estimated effect size and its precision (such as 95% confidence interval).
	For binary outcomes, presentation of both absolute and relative effect sizes is recommended.
15. Harms	All important harms or unintended effects in each group

*For the purpose of this study, our original 15 point OQRS was reduced to a 14 point score because one of the 15 items we initially assessed for was “objectives” and this was assessed as part of our PICOT score instead.

^a^The descriptions of the reporting items were taken directly from the CONSORT 2010 [16].

### Rating of overall reporting quality and key methodological items

In our previous study [1] we defined the quality of reporting as the extent which the rationale, design, conduct and analysis of each RCT evaluated was reported [1]. We did this by using 15 items from the 2010 CONSORT statement. The 15 items evaluated are reported in Table 1. These 15 items were selected for in our previous study [1] because the literature indicates that an absence in reporting these items is associated with a higher level of bias [16]. CONSORT items under “discussion” were excluded as we deemed them to be too subjective to assess. An OQRS, with 15 items in total (possible score from 1 to 15), was established. For scoring purposes, each of the 15 items was scored 1 if it was reported appropriately and 0 if it was not evidently stated or completely not stated. This score was reduced to 14 for this study for the reason mentioned above.

Three methodological items, which are a part of the CONSORT statement, were also excluded from our 14 point OQRS for a separate assessment to create the KMIS. These three methodological items are: 1) appropriate concealment of allocation, 2) blinding, and 3) numbers analysed (or known as the intention-to-treat principle). They were assessed separately because they are highly important in avoiding bias and misrepresentation of the treatment effect estimates [30-35]. Each of the three mentioned methodological items were given a score of 1 if the method was appropriate and 0 if it was inappropriate or vaguely reported. A combined KMIS was calculated for each RCT by adding the scores of each methodological item with a possible KMIS ranging from 0 to 3 [1].

We considered allocation concealment to be done appropriately if centralized randomization, coded, numbered vehicles or sealed, opaque, and sequentially numbered envelopes were reported [36]. For blinding, in trials where there were no barriers to blind groups, at least two groups had to be explicitly reported as blinded to fulfil this item’s criteria. In instances where blinding was not feasible, we considered blinding to have been appropriately done if at least one group was unambiguously reported as blinded [3,37,38]. In our previous study [1], numbers analysed based on intention-to-treat (ITT) analysis was defined as a study that included all participants randomized into an RCT and the trials statistical analysis regardless if: 1) the intervention was administered, 2) if patients fulfilled the study entry criteria and 3) if patients withdrew from the trial, or there were treatment deviations from protocol [37,39-41]. The meaning of ITT has been interpreted differently by various RCTs, however the Cochrane Handbook has compiled evidence specifying that the definition we used averts a biased treatment effect and is a practical way to understand the effects of a clinical intervention [41,42].

As described previously, a number of predictor variables have been shown to have a positive association with the reporting quality of RCTs [3,6,11,15,16,19-23]. The four predictor variables we used were sample size, impact factor, funding reported and journal adoption of the CONSORT statement at the time of our data abstraction. This step, as already mentioned above, was done in our previous study that included the 23 RCTs used in this study [1]. Sample size was defined as number of patients randomized in each trial. The impact factor of the journal refers to an index number that is assigned to a journal that is catalogued with Thomson Reuter’s Journal Citation Reports. This index number is calculated by Thomson Reuters [43] and is a ratio that describes the frequency that an “average article” in a particular journal has been cited in a specific year or period of time [43]. Funding reported was defined as funding that was mentioned and provided for the undertaking of the study. Journal adoption of the CONSORT statement at the time of our data abstraction was defined as the journal (within which that RCT is published) endorsing, enforcing or encouraging the use of the CONSORT statement for manuscript submissions.

### The rating of the research question in PICOT format

We identified one paragraph from the introduction or methods section that we found best stated the primary research question, hypothesis or objective. To accomplish this, reviewers read both the introduction and methods section careful to identify the paragraph which best described the aims of the study. That paragraph was then reread to see which PICOT components were mentioned. We chose one paragraph because according to the PICOT framework, a research question should be cohesive and put together in a single statement [25]. One paragraph should be sufficient to include all the components of a PICOT question. This enables the reader to clearly understand the intention behind the study without having to repeatedly sift through different sections of the paper to piece together the entire research question. In those paragraphs, we evaluated the framing of the research question, regardless if it was in the form of a research question, objective or hypothesis. We then evaluated if the 5 elements of a research question were present in that paragraph. The 5 elements were, the type of population or patient pertinent to the question, the intervention, the comparative intervention, the outcome of interest, and the time allocated for measuring the outcome or outcomes of interest (P, I, C, O and T respectively). We scored each PICOT element with a 1 if it was explicitly stated and 0 if it was absent. In the end we created a PICOT score with a potential score between 0 and 5 inclusively. This scoring system for the PICOT was used in a study done by Rios *et al.*[24]. The score represents how complete the primary research question is within the evaluated RCT. We decided to qualify a study as providing a structured research question only if the five elements (Complete PICOT) were present in the description of the primary research question, the study objectives or in a declared research hypothesis. RCTs that did not describe all 5 elements (Incomplete PICOT) were deemed to not have included a structured research question.

### Hypotheses

We hypothesized that higher PICOT scores will be associated with better quality of reporting as judged by the CONSORT completeness of reporting based on the OQRS and KMIS score system used in this study.

### Data abstraction

We used an Excel standardized data abstraction form to extract data from each article. Two reviewers (VJBD and SZ) were blinded to each other’s ratings and extracted data independently. When the research question was rated, the reviewers were blinded to the OQRS and score for the KMIS for each article that was done for a previous study [1]. Any disagreements were resolved through consensus. Kappa statistics were used to measure the inter rater agreement for each of the 5 PICOT elements of the research question. The Kappa statistics for the OQRS and the KMIS have been reported in our previous study [1].

### Statistical analysis

Categorical data were reported as number of counts. Each PICOT element was coded as a binary variable (1 = the element was clearly addressed in the research question and 0 = it was not clearly addressed in the research question). The number of RCTs that explicitly stated the PICOT element and the associated 95% confidence interval were calculated. The PICOT score was computed as the sum of the 5 individual elements and ranged from 0 to 5 inclusively. For the element that had a “zero” count or “full” counts, for instance when none of or all of the included trials reported that PICOT element, the 95% CI was calculated by adopting the rule of three [44]. This means that if none of *n* individuals within a PICOT element (i.e. one of the PICOT elements: P. I, C, O or T) showed the event that we were interested in, we could be 95% confident that the chance of this event occurring is at most 3 in *n*[45]. For the other PICOT elements, the 95% CI of the count was calculated by making the assumption that the number of RCTs that clearly stated the element followed a binomial distribution. The probability that an RCT had clearly stated the element was set to be the observed probability in the sample. The Cohen’s Kappa (κ) statistics were used to calculate the chance-adjusted agreement between the 2 raters for every PICOT element. Agreement was interpreted as poor if κ ≤ 0.2, fair if 0.21 ≤ κ ≤ 0.4, moderate if 0.41 ≤ κ ≤0.6, substantial if 0.61 ≤ κ ≤ 0.8 and good if κ > 0.8 [45].

A multivariable regression analysis, where the OQRS score was the outcome variable, was done to determine if a higher PICOT score was independently associated with a better OQRS. We included funding reported, journal adoption of the CONSORT statement at the time of data abstraction, sample size and the impact factor of the journal the RCTs were from as covariates for the OQRS. Here these variables were adjusted as covariates in the model. The sample size was transformed by the logarithm function with base 10 to improve interpretability. The OQRS (discrete, ranged from 0–14) was assumed to follow a Poisson distribution. The incidence rate ratio (IRR) was used to state the results of the analysis. The same method was used for the regression analyses with the KMIS as the outcome variable. Variables were considered to be statistically significant at alpha = 0.05. Regression analyses were also conducted to explore which individual PICOT elements were more associated with a better OQRS with adjustment of confounding variables. We did not adjust the overall level of significance for multiple testing because these analyses were primarily exploratory [46]. SPSS© 19.0.0.0 (IBM Corporation, 2010) was used to perform the statistical analyses.

## Results

### Rating of the research question using the PICOT framework

For the rating of the individual elements of the PICOT research question, the κ inter-rater agreement estimate using Cohen’s Kappa varied from 0.623 to 1. The median PICOT score was 3 (IQR = 1). The percentage of articles that described each PICOT element of a research question is in Table 2. Patients, intervention and comparators were often described well. A minority of the articles described the study’s time frame and less than 55% of the studies adequately described outcomes within the research question in that there was mention of the primary and secondary outcomes of the study. A complete PICOT structured research question was present in 2 out of the 23 RCTs evaluated.

**Table 2:** Frequency of description of each PICOT element

**Element of the research question**	**All articles (n = 23)**	
	**Count**	95% CI*
**P: Patients**	21	(18, 23)
**I: Invention**	23	(15, 23)
**C: Comparator**	19	(15, 22)
**O: Outcome**	12	(7, 17)
**T: Timeframe**	4	(1, 8)
**Complete PICOT**	2	(0, 5)

CI: Confidence Interval.

*For item that has non-zero event, the 95% CI was approximated by assuming the number of events followed a Binomial distribution; for item that has zero event, the 95% CI was approximated by the rule of three [44].

### Factors associated between PICOT framing of a research question and quality of reporting and key methodological item reporting

Table 3 and 4 display the results of the multivariable analysis for the factors associated with the OQRS and the KMIS, respectively. After adjusting for sample size, impact factor, journal adoption of the CONSORT statement at the time of our data abstraction and funding reported, a higher PICOT score was not significantly associated with a higher OQRS (Table 3) or KMIS (Table 4) in the multivariable analysis. The model however did show a trend for the PICOT score and OQRS but not KMIS. At each point or one unit increase in the PICOT score there was a detected 26.7% increase in the number of quality of reporting items discussed, on average. We did not find a significant association between any one of the PICOT items and the outcomes OQRS and KMIS (Table 5). In addition, each of the four covariates: sample size, impact factor, journal adoption of the CONSORT statement, and funding reported were not significantly associated with the outcomes OQRS and KMIS in the univariate analyses. The results of the univariate analysis are reported in Table 3 and Table 4.

**Table 3:** **Association between PICOT score and Overall Quality of Reporting Score (OQRS)**^**a**^

	**Univariate analysis**			**Multi-variable analysis**		
**Predictor variable**	**IRR**	**95% CI**	***P *** **value**	**IRR**	**95% CI**	***P *** **value**
PICOT	1.159	(0.835, 1.608)	0.378	1.267	(0.984, 1.630)	0.066
Sample Size^b^	0.998	(0.991, 1.006)	0.681	0.546	(0.199, 1.498)	0.240
Impact Factor	0.949	(0.744, 1.209)	0.67	0.915	(0.690, 1.212)	0.535
Journal adopted CONSORT Statement at the time of data collection.	0.898	(0.646, 1.248)	0.521	1.063	(0.721, 1.568)	0.757
Funding reported	1.241	(0.817, 1.887)	0.312	1.147	(0.722, 1.823)	0.562

CI: Confidence Interval; IRR: Incidence Rate Ratio.

^a^Maximum Possible Score for the Overall Quality of Reporting Score = 14.

^b^The sample size variable was log(10) transformed. The value is an expression of the change in the average of the OQRS due to one unit increase in sample size in the log scale.

**Table 4:** **Association between PICOT score and Key Methodological Items Score (KMIS)**^**a**^

	**Univariate analysis**			**Multi-variable analysis**		
**Predictor Variable**	**IRR**	**95% CI**	***P *** **value**	**IRR**	**95% CI**	***P *** **value**
PICOT	1.007	(0.987, 1.027)	0.492	1.061	(0.515, 2.188)	0.872
Sample Size^b^	1.746	(0.677, 4.504)	0.249	2.139	(0.126, 36.243)	0.598
Impact Factor	1.601	(0.784, 3.269)	0.196	2.037	(0.739, 5.614)	0.169
Journal adopted CONSORT Statement at the time of data collection.	0.909	(0.359, 2.303)	0.841	0.528	(0.147, 1.900)	0.329
Funding reported	2.051	(0.731, 5.754)	0.172	1.697	(0.533, 5.408)	0.371

CI: Confidence Interval; IRR: Incidence Rate Ratio.

^a^Maximum Possible Score for the Key Methodological Items Score = 3.

^b^The sample size variable was log(10) transformed. The value is an expression of the change in the average of the KMIS due to one unit increase in sample size in the log scale.

**Table 5:** **Association between individual PICOT items and the outcomes (Overall Quality of Reporting Score (OQRS)**^**a **^**and the Key Methodological Items Score (KMIS)**^**b**^**)**

**Overall quality of reporting score (OQRS)**^**a**^			
**Predictor variable**	**IRR**	**95% CI**	***P *** **value**
**Population**			
P – Population	1.427	(0.566, 3.593)	0.451
Sample size^c^	0.737	(0.287, 1.894)	0.527
Impact factor	0.939	(0.714, 1.236)	0.655
Journal adopted CONSORT Statement at the time of data collection.	0.954	(0.665, 1.368)	0.796
Funding reported	1.267	(0.818, 1.962)	0.290
**Control**			
C - Control	1.459	(0.855, 2.490)	0.166
Sample size^c^	0.679	(0.264, 1.750)	0.423
Impact factor	0.921	(0.699, 1.212)	0.556
Journal adopted CONSORT Statement at the time of data collection.	0.871	(0.604, 1.256)	0.458
Funding reported	1.448	(0.910, 2.304)	0.119
**Outcome**			
O - Outcome	1.099	(0.726, 1.664)	0.655
Sample size^c^	0.753	(0.293, 1.939)	0.557
Impact factor	0.915	(0.691, 1.212)	0.536
Journal adopted CONSORT Statement at the time of data collection.	0.972	(0.648, 1.458)	0.891
Funding reported	1.236	(0.762, 2.008)	0.391
**Timeframe**			
T- Timeframe	1.320	(0.818, 2.131)	0.256
Sample size^c^	0.611	(0.215, 1.733)	0.354
Impact factor	0.935	(0.708, 1.236)	0.638
Journal adopted CONSORT Statement at the time of data collection.	0.987	(0.676, 1.440)	0.945
Funding reported	1.234	(0.790, 1.928)	0.355
**Key methodological items score (KMIS)**^**b**^			
**Population**			
P- Population	0.726	(0.085, 6.216)	0.770
Sample size^c^	2.548	(0.194, 33.401)	0.476
Impact factor	2.032	(0.732, 5.641)	0.173
Journal adopted CONSORT Statement at the time of data collection.	0.496	(0.136, 1.812)	0.289
Funding reported	1.811	(0.624, 5.260)	0.275
**Control**			
C - Control	2.068	(0.386, 11.076)	0.396
Sample size^c^	1.674	(0.127, 22.148)	0.696
Impact factor	1.995	(0.732, 5.436)	0.177
Journal adopted CONSORT Statement at the time of data collection.	0.457	(0.127, 1.641)	0.230
Funding reported	2.130	(0.665, 6.825)	0.203
**Outcome**			
O - Outcome	0.948	(0.277, 3.245)	0.933
Sample size^c^	2.418	(0.189, 30.923)	0.497
Impact factor	2.017	(0.744, 5.470)	0.168
Journal adopted CONSORT Statement at the time of data collection.	0.507	(0.134, 1.919)	0.317
Funding reported	1.823	(0.506, 6.569)	0.359
**Timeframe**			
T - Timeframe	0.799	(0.187, 3.403)	0.761
Sample size^c^	2.858	(0.181, 45.059)	0.455
Impact factor	1.973	(0.725, 5.371)	0.184
Journal adopted CONSORT Statement at the time of data collection.	0.514	(0.151, 1.755)	0.288
Funding reported	1.872	(0.618, 5.668)	0.267

CI: Confidence Interval; IRR: Incidence Rate Ratio.

^a^Maximum Possible Score for the Overall Quality of Reporting Score (OQRS) = 14.

^**b**^Maximum Possible Score for the Key Methodological Items Score = 3.

^c^The sample size variable was log(10) transformed. The value is an expression of the change in the average of the OQRS due to one unit increase in sample size in the log scale.

No individual analysis of the Intervention (I) component of the PICOT score was done because all studies reported their intervention when they described their research question.

## Discussion

We assessed the use of the PICOT framing of a research question in 23 RCTs used within a meta-analysis from 2010 assessing the use of FNB in improving analgesia outcome after total knee arthroplasty [2]. We found that the framing of the research question was usually incomplete, ambiguous and inconsistently written in either the introduction or methods section of the study. Sometimes parts of the research question were split between the introduction and methods section. Only 2 out of the 23 RCTs had a completely structured research question using the PICOT format. This finding is not uncommon as two studies have previously shown poor PICOT framing of research question among RCTs [24,25], where in one study a structured research question was present in only 33.7% of the reports assessed [24].

From our literature search this is the second study to look at the association between PICOT framing of a research question and the overall quality of reporting and reporting of key methodological items of RCTs. This paper is unique in that it reviews RCT literature in anesthesia and studies used in a meta-analysis. Our results did not show a significant association between the completeness of the PICOT framing of a research question and OQRS and KMIS using the alpha value we set for significance (set as p < 0.05). Although we would attribute this to our small sample size, the results should be interpreted with caution. We did however uncover trends and tried to make comparisons to other studies using PICOT framing or some analogous predictor variable for OQRS or KMIS. After a review of the literature one study was found that showed an association between PICOT framing of a research question with overall reporting quality and reporting of key methodological items and that reported findings in a statistical and numerical approach that could be used for a trend comparison [24]. We were also able to compare the sample size predictor variable from this study.

For the OQRS outcome variable there were a few trends noted. For PICOT framing of a research question (predictor variable) the direction, magnitude and range of the effect was similar to the comparator study [24]. For the predictor variable of sample size, our direction and magnitude of the effect was different, however our range from our 95% confidence interval (CI) encompassed the incidence rate ratio (IRR) of the comparator study. Again, as mentioned previously, this is likely attributed to our small sample size.

For the KMIS outcome variable we also found some trends. For both predictor variables, PICOT framing of a research question and sample size, the direction, magnitude and range of the effect was similar to the comparator study. Our results are promising because in spite of our limitation with our own RCT sample size we still could show trends indicating a positive association between proper and complete PICOT framing of a research question and OQRS but not KMIS.

The noted association with the completeness of the PICOT framing of a research question and the quality of reporting is important [24] as it suggests that the theoretical reasoning behind systematically structuring a research question has practical and tangible applications in improving study design and transparency in the actual reporting. A desirable research question is one that is framed to constructively aid knowledge acquisition [47]. The question’s framing itself plays an important role in methodically and systematically clarifying the thought process needed to assess required outcomes and the purpose and benefit of the question itself [47,48]. A structured research question is focused as questions too broad will likely lack methodological rigor [47,48]. The PICOT method is a good approach as its five constituents help to focus a question and incite thoughts about rigorous methodological design and the feasibility of answering the question [25,48,49].

### Limitations

It is important to note the various limitations in our study, some background on the effect these drawbacks have and any future improvements. One important limitation of our study is that there is no standard instrument to evaluate the quality of reporting of RCTs. The quality scores attained from our evaluation instrument are not validated. Most scales used to evaluate the quality of reporting have not been thoroughly developed or tested as a gold standard (external criterion) is needed in order to compare for validity and reliability of the scale developed [50]. Because a gold standard does not currently exist for reporting the quality of RCTs, scales used for this purpose of reporting quality are only assessed based on an accepted theoretical model [50,51]. There has not been a single scale shown at being superior at measuring the quality of reporting alluding that dissimilar attributes in reporting are probably being measured [52,53] or there is, as we infer, an element of subjectivity in the way the scale allows for assessment. Quality scores based on checklists, such as the ones in our study, may also be unreliable and could introduce bias [52,53]. Scores were shown to differ contingent on the scoring system that was used [52,53]. The benefit of using the evaluation instrument we used is that it is based on the CONSORT checklist criteria from the 2010 CONSORT statement and the statement itself is widely recognized by journals and editorial groups [18]. The items we assessed for in this study are founded on well recognized features expected in the reporting and conduct of RCTs [18]. Another potential limitation of this study is that some PICOT items may be directly related to the CONSORT statement items used to create the OQRS. For instance, PICOT items “Population”, “Intervention” and “Outcome” may be directly related to the CONSORT items “Participants”, “Intervention” and “Outcomes”. This may have a possible effect on the analysis. To address this possible effect we looked at the associations between the individual PICOT items and the outcomes used in this study (Overall Quality of Reporting Score (OQRS) and the Key Methodological Items Score (KMIS)). The results of this analysis are in Table 5. None of the potential associations with the individual PICOT items were statistically significant—which may also be due to low statistical power. In the future it might be best to assess each individual item in our checklists for the OQRS and KMIS against the completeness of the PICOT structured research question [17] and substantially increase our sample size of RCTs to improve the power needed to detect a difference. Lastly, another possible limitation is the fact that the RCTs that we evaluated mostly come from anesthesia specialized journals, which may appear to reduce the generalizability of our findings. However, the generalizability would not be affected by this because the relationship between PICOT and the quality of reporting is not expected to be different depending on the field from which the RCTs come from. The aim of our study was to assess association between PICOT framing of a research question and OQRS and KMIS within a meta-analysis that has the potential to inform clinical practice. To increase the generalizability, a study of similar design with a much larger sample size of RCTs would be needed. Regardless of these noted limitations, this study brings value because it shows that proper and clear PICOT framing of a research question is still limited. We also showed that we have internal validity due to our good inter-rater reliability correlation between two reviewers who independently assessed the quality of reporting, key methodological items and PICOT framing of the research question. Our results should be interpreted with caution as a small sample size was used and we did not adjust for multiple testing in our analysis as our analyses were only exploratory.

## Conclusions

This study showed that PICOT framing of a research question in anesthesia related RCTs are incompletely structured with only two RCTs having a completely structured research question out of the 23 RCTs assessed. Although we did not find a statistically significant association between the completeness of the PICOT structured research question and OQRS and KMIS, our comparison with another study [24] showed strong trends between PICOT and OQRS but not KMIS. We are aware that our sample size is small and that cautious interpretation of the results should be made due to this. Poor overall quality of reporting does not mean there is poor methodological rigor within RCTs as some or even many features of a trial may not be adequately reported unless a protocol is assessed in conjunction. However, protocols are not as easily accessible and researchers when publishing should be aware that the publication of a study is a surrogate when assessing for study quality. The outcome of this study for researchers is that the proper and complete PICOT structure of a research question is an important attribute to all studies as it helps to indirectly improve reporting by initiating the thought process needed for thorough study design and management.

## Abbreviations

CI: Confidence interval; CONSORT: Consolidated Standards of Reporting Trials; FNB: Femoral Nerve Block; IRR: Incidence Rate Ratio; IQR: Inter-Quartile Range; KMIS: Key Methodological Item Score; RCT: Randomized Controlled Trial; OQRS: Overall Quality of Reporting; PICOT: Population, Intervention, Comparator, Outcome, Timeframe; SPSS: Statistical Package for the Social Sciences.

## Competing interests

The authors declare that they have no competing interests.

## Authors’ contributions

VBD was involved with the conception of the study, participated in the study design, the acquisition of data, helped to perform the statistical analysis, made substantial contributions to the interpretation of the data, and drafted the manuscript. SZ was substantially involved with data acquisition, and critical revisions to the paper. CY made substantial contributions to the statistical design, statistical analysis and interpretation of data and helped critically revise the manuscript. LT was involved with the conception of the study and made substantial contributions to the statistical analysis, interpretation of data and critically revised the manuscript. JP, LH, AA and YM were involved with contributing data and helped draft and edit the final manuscript. All authors read and approved the final manuscript.

## Pre-publication history

The pre-publication history for this paper can be accessed here:

http://www.biomedcentral.com/1471-2253/13/44/prepub
